# Weighted Gene Co-Expression Network Analysis to Identify Potential Biological Processes and Key Genes in COVID-19-Related Stroke

**DOI:** 10.1155/2022/4526022

**Published:** 2022-05-09

**Authors:** Gengyu Cen, Liuyu Liu, Jun Wang, Xue Wang, Shijian Chen, Yiting Song, Zhijian Liang

**Affiliations:** Department of Neurology, The First Affiliated Hospital of Guangxi Medical University, No. 6 Shuangyong Road, Nanning, 530021 Guangxi, China

## Abstract

The purpose of this research was to explore the underlying biological processes causing coronavirus disease 2019- (COVID-19-) related stroke. The Gene Expression Omnibus (GEO) database was utilized to obtain four COVID-19 datasets and two stroke datasets. Thereafter, we identified key modules via weighted gene co-expression network analysis, following which COVID-19- and stroke-related crucial modules were crossed to identify the common genes of COVID-19-related stroke. The common genes were intersected with the stroke-related hub genes screened via Cytoscape software to discover the critical genes associated with COVID-19-related stroke. Gene Ontology (GO) and Kyoto Encyclopedia of Genes and Genomes (KEGG) enrichment analysis for common genes associated with COVID-19-related stroke, and the Reactome database was used to annotate and visualize the pathways involved in the key genes. Two COVID-19-related crucial modules and one stroke-related crucial module were identified. Subsequently, the top five genes were screened as hub genes after visualizing the genes of stroke-related critical module using Cytoscape. By intersecting the COVID-19- and stroke-related crucial modules, 28 common genes for COVID-19-related stroke were identified. ITGA2B and ITGB3 have been further identified as crucial genes of COVID-19-related stroke. Functional enrichment analysis indicated that both ITGA2B and ITGB3 were involved in integrin signaling and the response to elevated platelet cytosolic Ca^2+^, thus regulating platelet activation, extracellular matrix- (ECM-) receptor interaction, the PI3K-Akt signaling pathway, and hematopoietic cell lineage. Therefore, platelet activation, ECM-receptor interaction, PI3K-Akt signaling pathway, and hematopoietic cell lineage may represent the potential biological processes associated with COVID-19-related stroke, and ITGA2B and ITGB3 may be potential intervention targets for COVID-19-related stroke.

## 1. Introduction

Since December 2019, severe acute respiratory syndrome coronavirus 2- (SARS-CoV-2-) induced coronavirus disease 2019 (COVID-19) has been causing significant morbidity and mortality around the world [1, 2]. According to a World Stroke Organization panel review, the risk of ischemic stroke increases by approximately 5% during COVID-19 [3]. Furthermore, when compared to influenza, COVID-19 has a 7.6-fold increase in the risk of cerebrovascular complications [4]. Typically, patients develop symptoms of COVID-19, including respiratory symptoms and fever, with ischemic stroke beginning at 10 days after the onset of the respiratory disease [5].

One of the complications of COVID-19 is ischemic stroke [6], and COVID-19-related stroke may be caused by various pathophysiological mechanisms. Infection with SARS-CoV-2 leads to severe vascular inflammation with significant endothelial injury, resulting in unregulated coagulation activation and venous and arterial thromboembolism [7–9]. COVID-19 is linked to proinflammatory cytokines, which activate endothelial cells and monocytes, as well as tissue factor expression, resulting in coagulation activation and thrombin production. Natural anticoagulants cannot limit the circulation of free thrombin, which can activate platelets and cause thrombosis [7], thereby leading to ischemic stroke. The molecular biological mechanisms underlying these pathophysiology pathways, however, remain unknown.

Detection of changes in gene expression during SARS-CoV-2 infection using multiple functional genomic approaches can improve our understanding of the molecular mechanisms involved in COVID-19 [10]. However, analysis of gene differential expression focusses more on individual genes effects, while ignoring the interaction of genes in complex biological gene networks, and fails to establish the relationship between genes and illnesses [11]. Hence, investigating gene interactions at the system level could help better understand the SARS-CoV-2 infection dynamics and the molecular mechanisms that contribute to COVID-19. In addition, this challenge may be tackled via weighted gene co-expression network analysis (WGCNA), which is a new biological approach for assessing the interaction between phenotypes, genes, and networks and gives great sensitivity and system-level insight to small-fold or low-abundance variation genes [12]. Recently, WGCNA has become increasingly popular as a tool for translating expression data of gene into co-expression modules and identifying potential biological processes and essential genes [13]. The purpose of this research was to reveal the possible biological processes of COVID-19-related stroke by analyzing the shared biological processes in COVID-19 and ischemic stroke co-expression networks. Furthermore, we discovered essential genes common to COVID-19 and ischemic stroke that are possible targets for intervention in COVID-19-related stroke.

## 2. Materials and Methods

### 2.1. Data Collection and Processing

**Figure 1:**
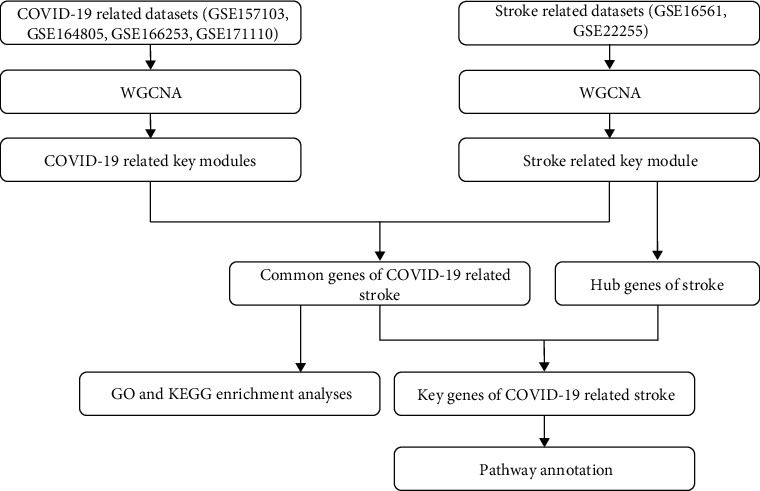
The process of data preparation and analysis. WGCNA: weighted gene co-expression network analysis; GO: Gene Ontology; KEGG: Kyoto Encyclopedia of Genes and Genomes.

Data preparation and analysis are illustrated in Figure 1. Four COVID-19 datasets (GSE157103, GSE164805, GSE166253, and GSE171110) and two stroke datasets (GSE16561 and GSE22255) were downloaded using the GEO public database (https://www.ncbi.nlm.nih.gov/geo/). The GSE157103 dataset comprised 26 non-COVID-19 controls and 100 patients with COVID-19 and is based on the GPL24676 Illumina NovaSeq 6000 (Homo sapiens) platform. The GSE164805 dataset, which is based on the GPL26963 Agilent-085982 Arraystar human lncRNA V5 microarray, comprised 10 patients with COVID-19 and 5 healthy controls. Next, the GSE166253 dataset, which is based on the GPL20795 HiSeq × Ten (Homo sapiens) platform, consisted of 16 patients with COVID-19 and 10 healthy controls. The GSE171110 dataset, which is based on the GPL16791 Illumina HiSeq 2500 platform (Homo sapiens), comprised 44 patients with COVID-19 and 10 healthy controls. Furthermore, the GSE16561 dataset, which was produced on the GPL6883 Illumina HumanRef-8 v3.0 expression beadchip platform, comprised 24 healthy controls and 39 patients with ischemic stroke. Finally, the GSE22255 dataset comprised 20 healthy controls and 20 patients with ischemic stroke and is based on the GPL570 Affymetrix Human Genome U133 Plus 2.0 platform. The samples of all the six datasets were composed of peripheral blood (Table 1).

To eliminate batch effects across all samples, the surrogate variable analysis (SVA, version 3.14) algorithm was utilized and presented the results of batch effect elimination through a PCA graph. Probe identifications were annotated as gene symbols and log2-transformation of the expression values. Subsequently, the COVID-19 datasets were combined, the common genes from the four datasets were collected, and a new gene expression profile for all samples was formed. The stroke datasets were combined, and the common genes were selected to form a new gene expression profile for all samples. Subsequently, we utilized the limma package's “normalizeBetweenArrays” function in R-Studio (version 4.1.0) to normalize the data. The average gene expression value was used if a gene has two or more expression levels. Finally, the org.Hs.eg.db package was utilized to reannotate the gene symbols of gene expression matrix as Entrez IDs for subsequent analysis.

### 2.2. WGCNA Construction and Disease-Related Key Module Identification

The coexpressed gene modules were discovered using the WGCNA algorithm in R-Studio (version 4.1.0) software. Sample clustering was performed to detect outliers, the “pickSoftThreshold” algorithm was utilized to select an appropriate soft threshold (*β*), and the scale independent > 0.8 biological significant scale-free network was obtained. The adjacency value was determined as the expression similarity between m and n of each gene pair: amn = power (Smn, *β*) = |Smn|*β* (the correlation coefficient absolute value between the gene expression matrix m and n was determined as Smn). To combat the influence of false or missing links between genes m and n, the adjacency matrix was transformed into a topological overlap matrix using the “TOMsimilarity” algorithm. Furthermore, the modules were also discovered using topological overlap matrix hierarchical clustering analysis and the Dynamic Branch-Cut method. The module eigengene values were determined using the “moduleEigengenes” algorithm, and the gene expression profiles of specific modules were summarized. Hierarchical clustering and Spearman correlation analyses were performed between the module eigengene values of modules and the clinical characteristics of samples, *P* values < 0.05 and correlation coefficient (*r*) > 0.3 are statistically significant [14], and these modules were identified as COVID-19- or stroke-related crucial modules.

### 2.3. Identification of Common and Crucial Genes in COVID-19-Related Stroke

By intersecting COVID-19-related crucial modules with stroke-related crucial modules, the common genes of COVID-19-related stroke were discovered. In addition, the STRING website (https://cn.string-db.org/) was employed to display its protein-protein interaction (PPI) relationships. Confidence score > 0.4 was statistically significant. The CytoHubba plug-in in Cytoscape (version 3.8.2) was applied to obtain the hub genes of stroke-related crucial modules. The crucial genes of COVID-19-related stroke were identified by crossing common COVID-19-related stroke genes with stroke-related hub genes.

### 2.4. Functional Enrichment and Pathway Analysis of Common and Crucial Genes in COVID-19-Related Stroke

The org.Hs.eg.db and clusterProfiler packages in R-Studio were applied to conduct GO and KEGG enrichment analyses of common genes in COVID-19-related stroke. GO describes the biological function of the overexpression of common genes in terms of biological processes, cellular components, and molecular function, whereas KEGG explains the common gene overrepresented pathways. The threshold of significant enrichment was adjusted to a *P* value < 0.05. The Reactome database (https://reactome.org/) is a peer-reviewed, open-access, open-source pathway database that is manually managed. In this study, the Reactome database was utilized to annotate and show the pathways implicated in key genes.

## 3. Results

### 3.1. Construction of WGCNA

**Figure 2:**
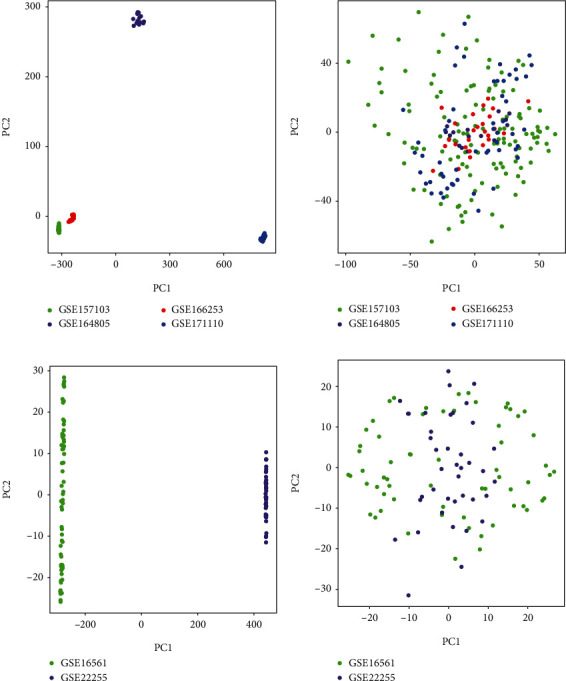
PCA of before and after elimination of batch effect for all samples from the COVID-19 and stroke datasets. (a, b) Represent the distribution of COVID-19 samples before and after elimination of batch effect, respectively. (c, d) Represent the distribution of stroke samples before and after eliminating the batch effect, respectively.

**Figure 3:**
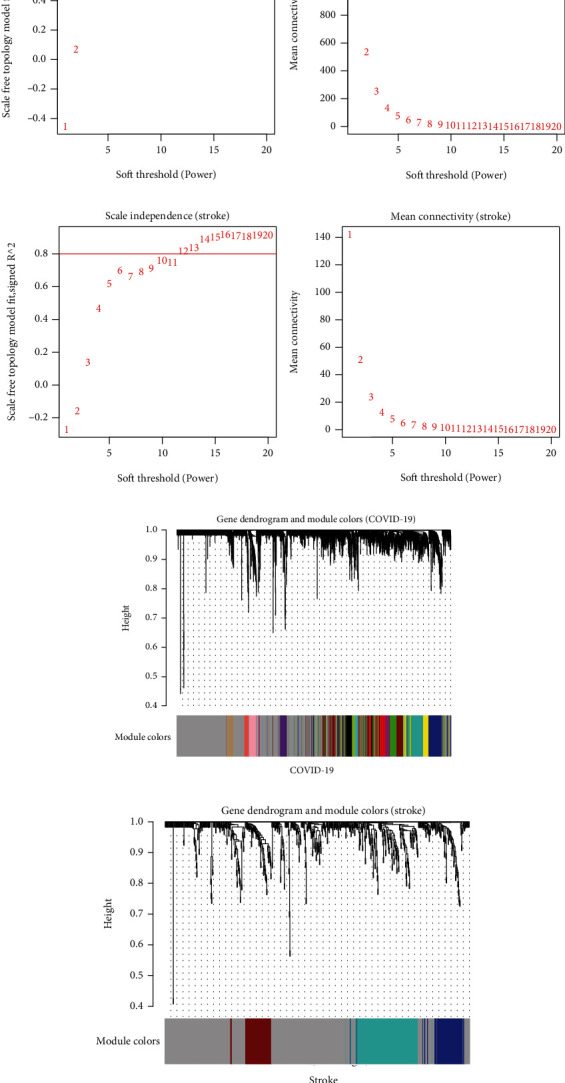
Construction of weighted co-expression network for COVID-19- and stroke-related datasets. (a, b) Network topology analysis of different soft threshold power. The effect of soft threshold power on the scale-free topology fit index is depicted in the left. The effect of soft threshold power on mean connectivity is depicted in the right. (c, d) Dendrograms of genes acquired by mean linkage hierarchical clustering. The allocation of modules decided by Dynamic Tree Cutting is displayed in the colored rows below the dendrogram.

Batch effects have been eliminated with SVA in all samples from the COVID-19 and stroke datasets, as shown in Figure 2. In the COVID-19- and stroke-related datasets, we used WGCNA to find coexpressed gene modules. The scale-free topological index was 0.8 when the soft thresholds for COVID-19 and stroke were 7 and 12, respectively, as illustrated in Figures 3(a) and 3(b). As a result, this network follows a power-law distribution and is more similar to the condition of a true biological network. Figures 3(c) and 3(d) show the derived gene dendrograms and their corresponding module colors.

### 3.2. Disease-Related Key Module Identification

**Figure 4:**
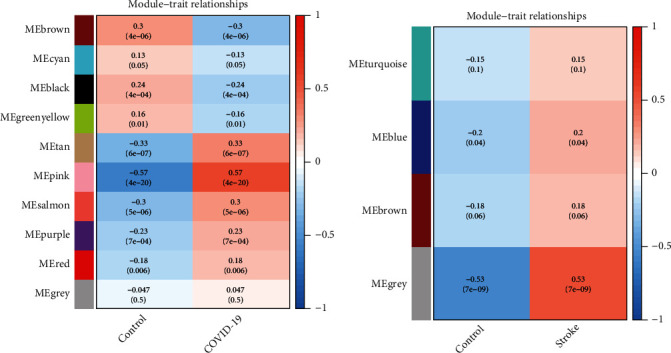
Module-trait relationships. (a, b) Each column represents a clinical feature (COVID-19 or stroke and control), and each row denotes an ME. The correlation coefficient and *P*value are contained in each cell. ME: module eigengene.

The hierarchical clustering and Spearman correlation analyses were used to calculate the correlations between each module and the two phenotypes (health and disease states), and the modules that were most relevant to the disease were identified. The heat maps of the correlation between values of module eigengene and clinical features showed that the pink (*r* = 0.57, *P* = 4*E* − 20) and tan (*r* = 0.33, *P* = 6*E* − 7) modules were highly positively related with COVID-19, while gray (*r* = 0.53, *P* = 7*E* − 9) modules were substantially positively related with stroke (Figures 4(a) and 4(b)). As a result, the pink and tan modules were recognized as COVID-19-related crucial modules, whereas the gray module was classified as stroke-related crucial module.

### 3.3. Identification of Common and Key Genes in COVID-19-Related Stroke

**Figure 5:**
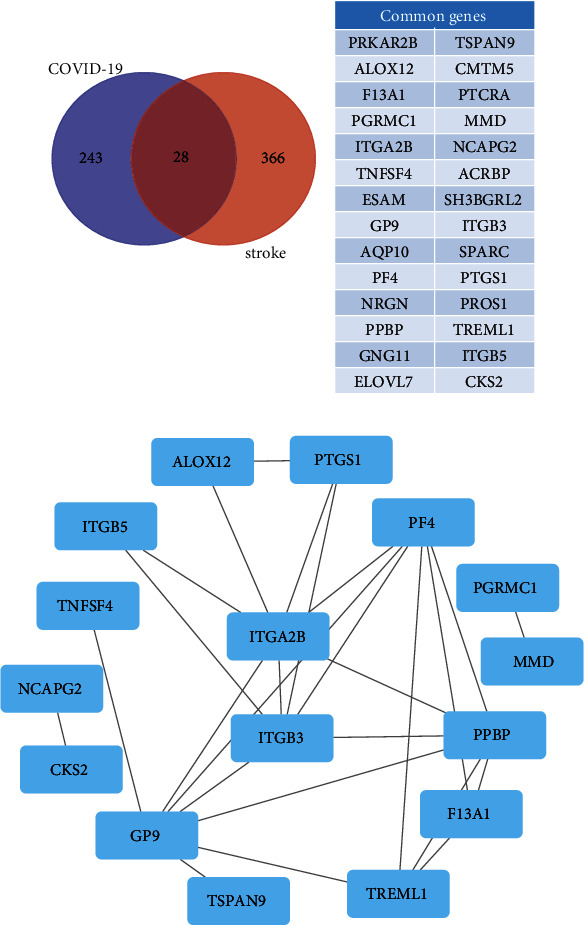
Identification of common genes of COVID-19-related stroke. (a) Venn diagram of common genes in COVID-19-related crucial modules and stroke-related crucial module. (b) A PPI network of common genes.

We initially identified the hub genes of the stroke-related crucial module to find the crucial genes of COVID-19-related stroke. The CytoHubba plug-in of the Cytoscape was utilized to visualize the genes of stroke-related crucial module, and the top five genes were selected as hub genes using EcCentricity as the ranking method (MPO, MMP9, ITGA2B, ITGB3, and RETN). Subsequently, by intersecting the COVID-19-related modules and the stroke-related module, the common genes were obtained. Twenty-eight common genes were discovered, as well as their protein-protein interactions (Figures 5(a) and 5(b)). In addition, ITGA2B and ITGB3 were obtained by crossing these common genes with the hub genes of stroke-related crucial module, suggesting that these two genes are more crucial than other common genes in the mechanisms of COVID-19-related stroke. As a result, ITGA2B and ITGB3 have been identified as important genes involved in COVID-19-related stroke.

### 3.4. Functional Enrichment Analysis on Common and Key Genes of COVID-19-Related Stroke

**Figure 6:**
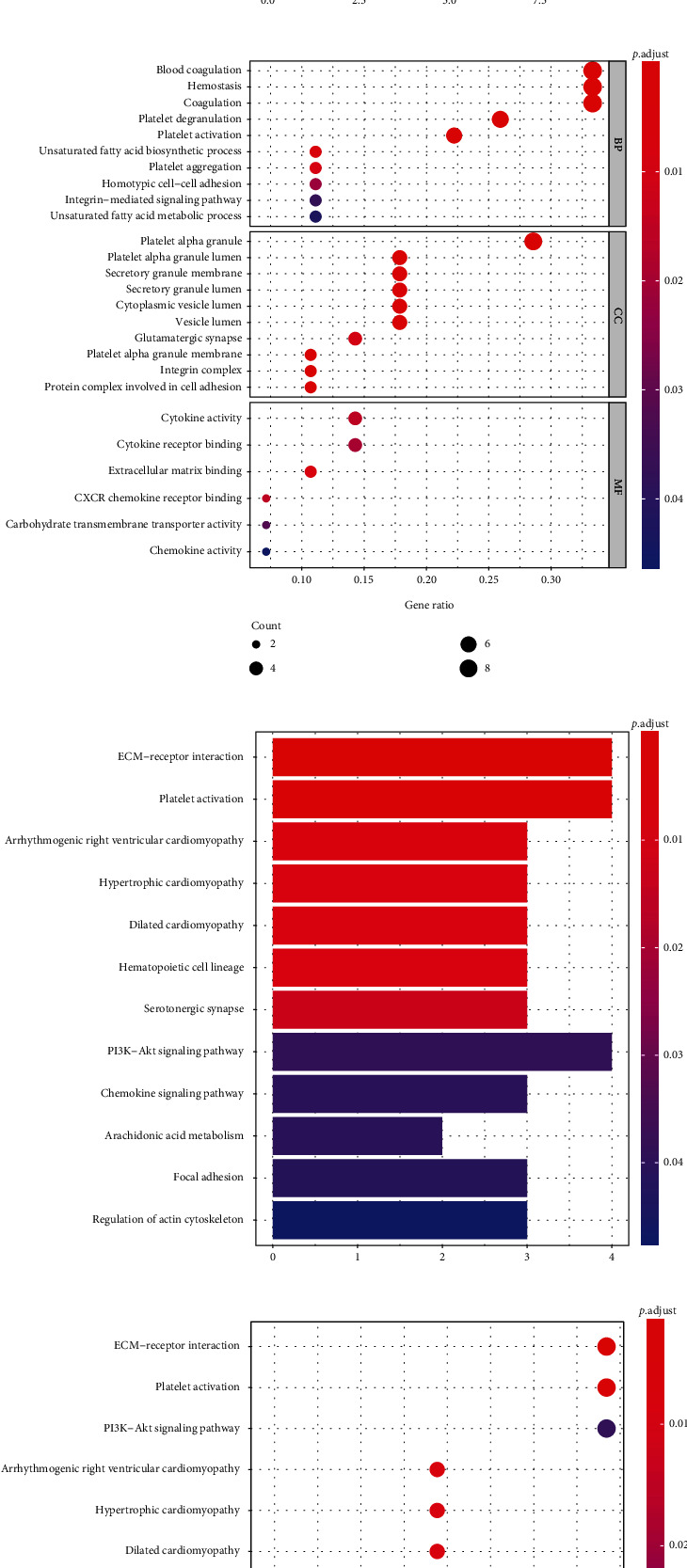
The results of functional analysis of common genes. (a, b) Gene Ontology (GO) enrichment analysis. (c, d) Kyoto Encyclopedia of Genes and Genomes (KEGG) enrichment analysis. BP: biological process; CC: cellular component; MF: molecular function.

**Figure 7:**
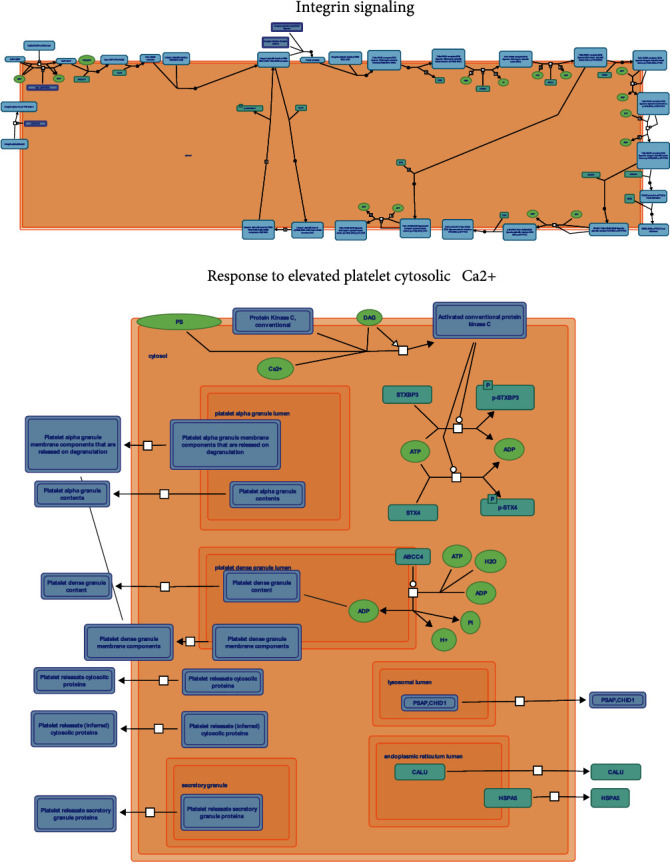
Pathway annotation of essential genes associated with COVID-19-related stroke. (a, b) The roles of essential gene regulatory pathways in detail.

We conducted GO and KEGG enrichment analysis on common COVID-19-related stroke genes, as well as pathway annotation of key COVID-19-related stroke genes, to discover the underlying molecular biological process of COVID-19-related stroke. With regard to the common genes associated with COVID-19-related stroke, the GO terms of molecular function showed that they were primarily enriched in CXCR chemokine receptor binding, carbohydrate transmembrane transporter activity, cytokine receptor binding, chemokine activity, cytokine activity, and extracellular matrix (ECM) binding. With regard to cellular components, the genes were primarily enriched in the platelet alpha granule membrane, protein complex involved in cell adhesion, cytoplasmic vesicle lumen, vesicle lumen, platelet alpha granule, integrin complex, secretory granule lumen, glutamatergic synapse, and secretory granule membrane. With regard to biological processes, the common genes were primarily enriched in platelet degranulation, blood coagulation, hemostasis, platelet activation, unsaturated fatty acid biosynthetic processes, platelet aggregation, homotypic cell-cell adhesion, integrin-mediated signaling pathways, and unsaturated fatty acid metabolic processes (Figures 6(a) and 6(b)). KEGG terms were enriched in platelet activation, ECM-receptor interaction, chemokine signaling pathway, hematopoietic cell lineage, serotonergic synapse, hypertrophic cardiomyopathy, PI3K-Akt signaling pathway, arrhythmogenic right ventricular cardiomyopathy, dilated cardiomyopathy, arachidonic acid metabolism, and focal adhesion (Figures 6(c) and 6(d)). Both ITGA2B and ITGB3 were involved in integrin signaling and the response to elevated platelet cytosolic Ca^2+^ levels (Figures 7(a) and 7(b)).

## 4. Discussion

The WGCNA package was used in this research to investigate probable biological processes and key genes associated with COVID-19-related stroke. Twenty-eight common genes were found to be present in both COVID-19- and stroke-related crucial modules, indicating that they are the most likely to have a biological function in COVID-19-related stroke. Using GO enrichment analysis, we discovered that the common genes were mostly associated with extracellular matrix binding, chemokine activity, cytokine receptor binding, cytokine activity, CXCR chemokine receptor binding (MF), platelet alpha granules, integrin complexes, protein complexes involved in cell adhesion (CC), platelet degranulation, blood coagulation, hemostasis, platelet activation, platelet aggregation, homotypic cell-cell adhesion, and the integrin-mediated signaling pathway (BP), all of which play a role in the formation and function of megakaryocytes and platelets [15–17]. These common genes were primarily enriched in platelet activation, ECM-receptor interaction, the PI3K-Akt signaling pathway, and hematopoietic cell lineage, according to the findings of KEGG enrichment analysis. Most crucially, ITGA2B and ITGB3 were shown to be the hub genes for key stroke-related modules, suggesting that they play critical roles in COVID-19-related stroke. According to functional annotation, through their participation in the regulation of integrin signaling and response to elevated platelet cytosolic Ca^2+^, the key genes play a role in COVID-19-related stroke.

Infection with the SARS-CoV-2 virus results in COVID-19 [18]. Although infection with SARS-COV-2 can cause acute respiratory distress syndrome, recent studies indicate patients with COVID-19 also suffer from thrombosis and multiple organ failure, such as ischemic stroke and myocardial infarction, and thrombosis occurs in 20–30% of patients with severe COVID-19 infection [19]. Severe COVID-19 patients are mostly found in men aged ≤55 years, but there is no gender difference in the incidence of COVID-19 in men and women aged over 55 years, namely, postmenopausal women. Normal levels of estrogen in women have a protective effect. But estrogen replacement therapy may increase the risk of thromboembolism by increasing platelet adhesion to vascular endothelial cells [20–22]. Accumulating evidence suggests that endothelial and platelet activation plays key roles in thrombosis [23, 24]. Since the discovery of viral inclusions in endothelial cells, research suggests that damage and activation of endothelial cell might lead to platelet activation and subsequent coagulopathy [25, 26]. The activity and antigen of Von Willebrand factor (vWF) and factor VIII were considerably elevated in patients with COVID-19, indicating an inflammation-mediated endothelial activation procoagulant state [27]. Endothelial cell dysfunction and exocytosis of abnormally large vWF multimers and platelet activation may result from direct viral invasion or indirect complement-mediated activation, resulting in microthrombogenesis [28, 29]. Recent studies have shown that the SARS-COV-2 spike protein 1 (S1) found in serum of COVID-19 patients can activate vascular endothelial cells and promote leukocyte adhesion. Meanwhile, S1 also leads to abundant deposition of C3 and C5b-9 on microvascular endothelial cells. The production of C3a and C5a further amplifies S1-induced complement activation. This eventually leads to increased platelet aggregation on the endothelial cells of microvessels [30]. Platelets, in addition to their conventional involvement in thrombosis and hemostasis, also play important roles in inflammatory and immunological processes [31–33], which indirectly influence immune responses by the release of antimicrobial peptides and cytokines and directly amplify immune responses by interacting with lymphocytes, monocytes, and neutrophils once pathogens have been detected [34]. Immunothrombosis, a prothrombotic response, is commonly related to inflammatory and infectious diseases, which usually happens when platelets are activated in response to invading pathogens. Immunothrombosis can cause detrimental immunological and hemostatic processes that contribute to vascular thrombosis, organ failure, and death in patients [35]. Patients with serious COVID-19 complications have similar hemostatic abnormalities related to disseminated intravascular coagulation and sepsis, contributing to a higher risk of thrombosis [36]. A prominent characteristic of acute ischemic stroke is platelet activation. Therefore, platelet activation may promote hypercoagulability and induce ischemic stroke in patients with COVID-19, which is a potential biological process of COVID-19-related stroke.

The phosphatidyl-inositol-3-kinase (PI3K)/AKT pathway is an essential signaling pathway triggered by various stimuli, including ECM proteins, integrins, growth factors, and cytokines, and controls various biological processes and different aspects of cell survival, including platelet activation [37, 38]. In addition, the pathway activates a variety of transcription factors to induce platelet production [15]. Platelet receptors such as GPIB-IX-V and integrin, which regulate platelet activation and hematopoiesis, also activate the PI3K-Akt pathway [38]. SARS-CoV-2 modulates platelet activation and thrombosis by overactivating the PI3K/AKT pathway [39, 40]. Tissue factor, fibrinogen, thrombin, angiotensin II, and inflammatory cytokines, such as tumor necrosis factor- (TNF-) *α*, transforming growth factor- (TGF-) *β*, interleukin- (IL-) 1, and IL-6, are elevated in patients with COVID-19, which activates the PI3K-Akt signaling pathway and induces platelet activation and formation, mediating a hypercoagulable state of blood during COVID-19 infection [40–43]. ECM is an acellular structure that provides tissue stiffness and cohesiveness, and bone marrow ECM is essential for normal hematopoiesis. Laminin, fibronectin, collagen, and fibrinogen are among the proteins contained in bone marrow ECM. In addition, different ECM components have different regulatory effects on platelet formation [17]. For example, platelet formation is facilitated by type III and IV collagens via the PI3K/Akt signaling pathway [44]. Fibrinogen binds to *α*IIb*β*3 and stimulates the production of proplatelet and platelet release [45]. In this study, we found that common genes for COVID-19-related stroke are enriched in platelet activation, ECM-receptor interaction, PI3K-Akt signaling pathway, and hematopoietic cell lineage, suggesting that these pathways may comprise the underlying biological processes for COVID-19-related stroke.

The ITGA2B gene encodes *α*IIb, which is expressed only in hematopoietic progenitors, platelets, and megakaryocytes, whereas ITGB3 encodes *β*3. In addition, fibrinogen receptor *α*IIb*β*3, which is an integrin, is formed by the two proteins [46]. Integrin *α*IIb*β*3 is a heterodimeric receptor abundantly expressed in platelet plasma membranes, and its activation and binding to soluble ligands (mainly fibrinogen, as well as vWF and fibronectin) are required for platelet activation, adhesion, and aggregation [38, 47, 48]. The *α*IIb*β*3 integrin has a modest affinity for ligands in resting platelets [49–51]. Inside-out cellular signaling, triggered by soluble agonists or subcutaneous matrices, generates conformational changes in integrin *α*IIb*β*3 during platelet activation, which allow for binding with fibrinogen with high affinity, acting as a “bridge” for additional activated platelets and ultimately establishing platelet plugs [49]. This signaling pathway is notable for the involvement of protein kinase C and PI3K. Outside-in signaling then causes cytoskeletal transformation, platelet granule production, stability, and clot retraction, all of which help solidify the fibrin clot [50, 51]. Talins are essential integrin activation regulators, and elevated intracellular concentrations of calcium result in conformational changes in the extracellular domains of integrin *α*IIb*β*3 subunits induced by talins, allowing their ligand binding sites to bind to fibrinogen molecules and facilitate platelet activation [50, 51]. In the present study, ITGA2B and ITGB3 were identified as key genes for COVID-19-related stroke, and these biological processes were closely associated with COVID-19-related stroke. A previous study indicates that platelet activation in patients with COVID-19 is dependent on the regulation of integrin *α*IIb*β*3 [52]. Bongiovanni et al. found that the levels of platelet activation markers p-selectin and integrin *α*IIb*β*3 were significantly increased in patients with COVID-19, suggesting an overactivated platelet phenotype during infection with SARS-CoV-2. This can cause a hypercoagulable state of COVID-19 and may have an impact on progression of disease. In patients with COVID-19, higher surface expression of integrins and adhesive proteins plays a crucial role in accelerating platelet aggregation and diffusion of fibrinogen and collagen [53]. We annotated the functions of ITGA2B and ITGB3, showing that they regulate integrin signaling and respond to elevated platelet cytosolic Ca^2+^ levels. Therefore, we speculated that ITGA2B and ITGB3 are likely to act in COVID-19-related stroke by participating in integrin signaling and in the response to elevated platelet cytosolic Ca^2+^, thus regulating platelet activation, ECM-receptor interaction, PI3K-Akt signaling pathway, and hematopoietic cell lineage. However, further experimental researches are needed to validate the regulatory mechanisms of these pathways and genes.

## 5. Conclusions

A total of 28 common genes may be involved in the mechanisms of COVID-19-related stroke through their involvement in platelet activation, ECM-receptor interaction, PI3K-Akt signaling pathway, and hematopoietic cell lineage. Additionally, ITGA2B and ITGB3 may be promising intervention targets for COVID-19-related stroke.

## Figures and Tables

**Table 1:** Data collection.

Condition	Tissue	GEO dataset	Platform	Number of samples
COVID-19	Peripheral blood	GSE157103	GPL24676	126
COVID-19	Peripheral blood	GSE164805	GPL26963	15
COVID-19	Peripheral blood	GSE166253	GPL20795	26
COVID-19	Peripheral blood	GSE171110	GPL16791	54
Ischemic stroke	Peripheral blood	GSE16561	GPL6883	63
Ischemic stroke	Peripheral blood	GSE22255	GPL570	40

## Data Availability

All data from the GSE157103, GSE164805, GSE166253, GSE171110, GSE16561, and GSE22255 datasets are available at the GEO database.

## Supplementary Materials

Supplementary Materials Figure S1: a PPI network of common genes of COVID-19-related stroke. Figure S2: the common genes of COVID-19-related stroke intersected with the stroke-related hub genes to identify the crucial genes of COVID-19-related stroke.
