# Supplementary figures and images for: Weighted Gene Co-Expression Network Analysis to Identify Potential Biological Processes and Key Genes in COVID-19-Related Stroke
Source: Oxid Med Cell Longev. 2022 May 9;2022:4526022. doi: 10.1155/2022/4526022 (PMC9088964; doi:10.1155/2022/4526022)

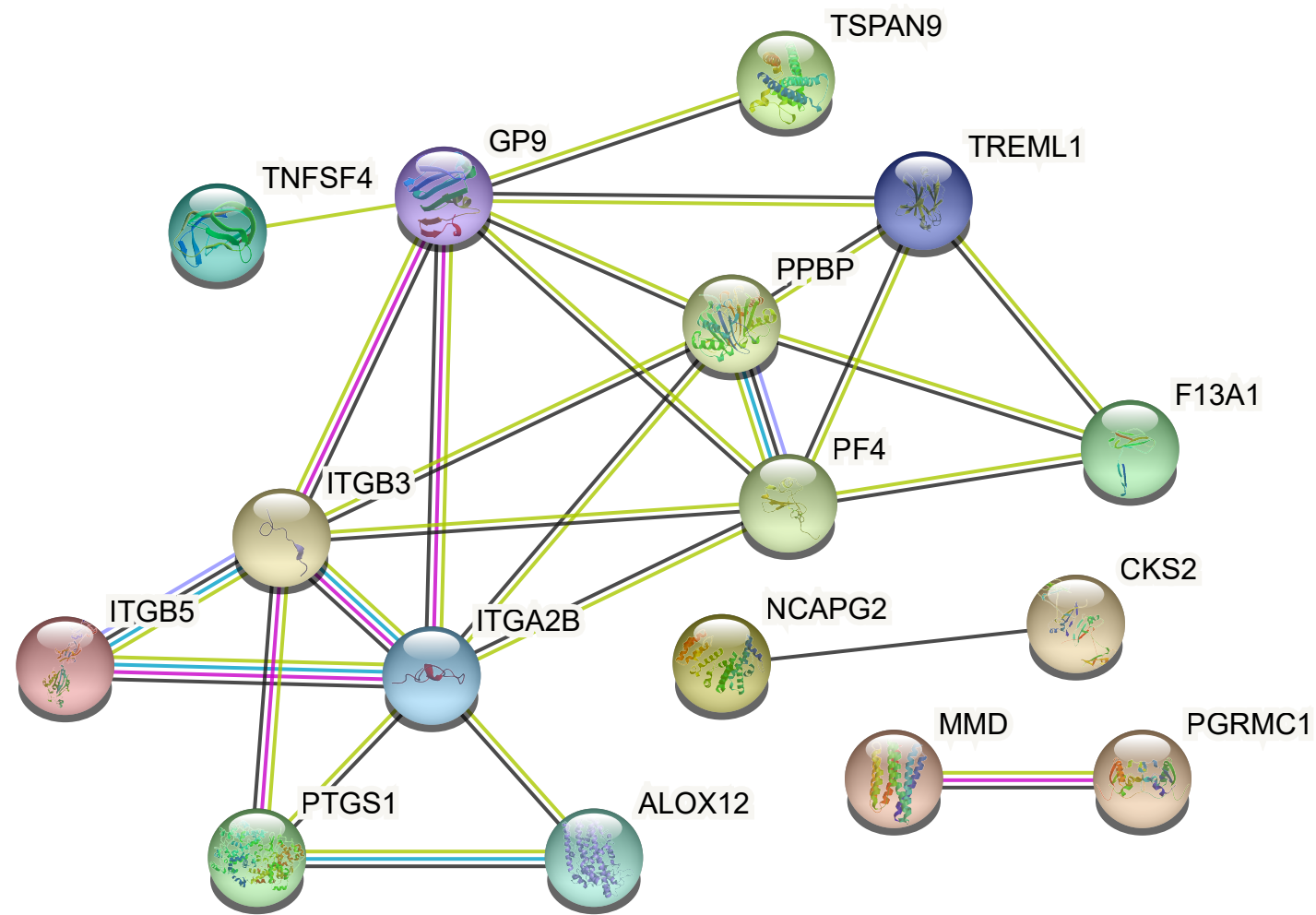

Supplement: Supplementary Materials — Figure S1: a PPI network of common genes of COVID-19-related stroke. Figure S2: the common genes of COVID-19-related stroke intersected with the stroke-related hub genes to identify the crucial genes of COVID-19-related stroke. [file 4526022.f1.zip › Supplementary Figure 1.pdf]

common

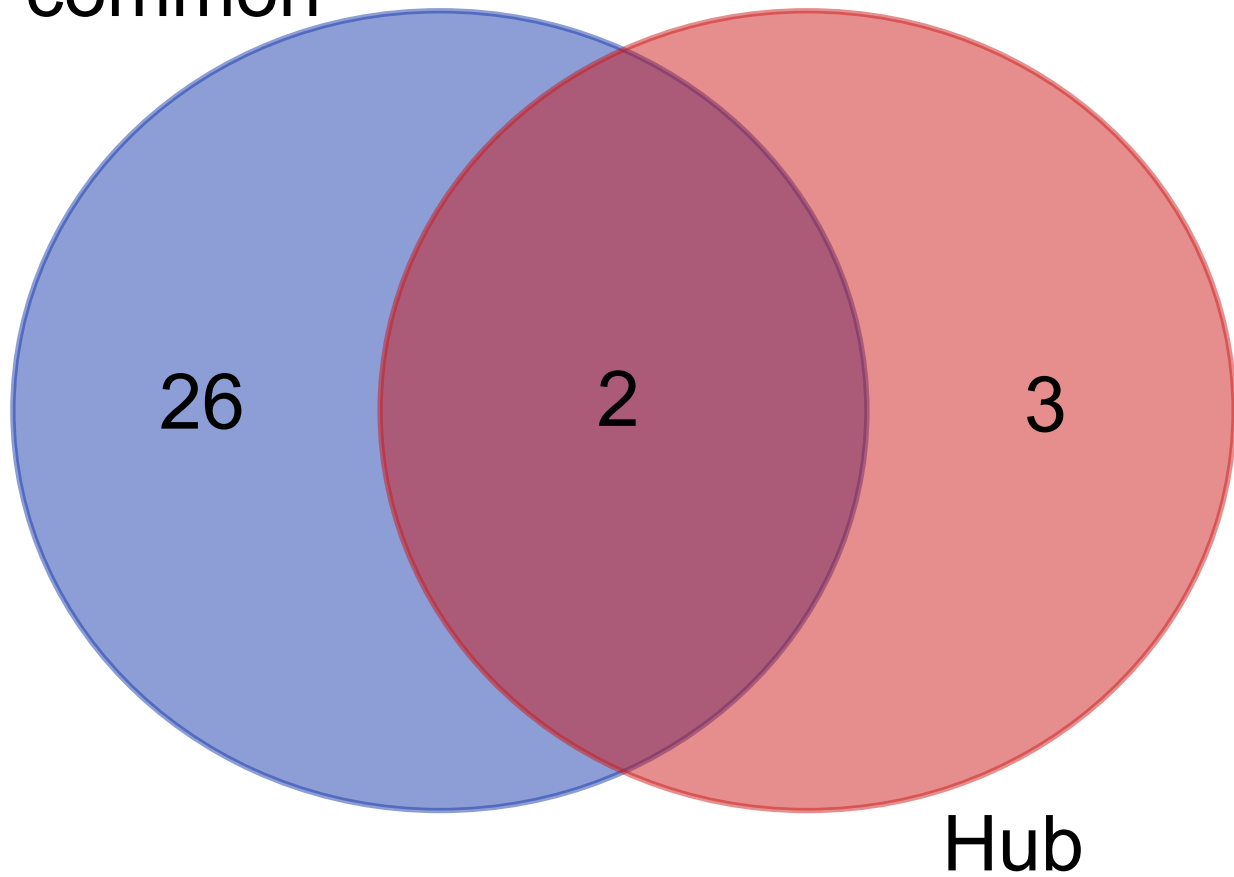

Hub

Supplement: Supplementary Materials — Figure S1: a PPI network of common genes of COVID-19-related stroke. Figure S2: the common genes of COVID-19-related stroke intersected with the stroke-related hub genes to identify the crucial genes of COVID-19-related stroke. [file 4526022.f1.zip › Supplementary Figure 2.pdf]
